# Visual Dependence and Fall Risk in Dementia: Implications for Sensory Re‐Weighting Strategies

**DOI:** 10.1111/ggi.70378

**Published:** 2026-01-29

**Authors:** Yi‐Ching Chu, Chao‐Chun Huang

**Affiliations:** ^1^ Department of Ophthalmology Far Eastern Memorial Hospital New Taipei City Taiwan; ^2^ Department of Physical Medicine and Rehabilitation Far Eastern Memorial Hospital New Taipei City Taiwan; ^3^ Department of Physical Therapy and Assistive Technology National Yang Ming Chiao Tung University Taipei Taiwan

To the Editor,

We read with great interest the article by Nakai et al. [1], which elucidated the significance of visual correction of balance—quantified by the Romberg quotient—in care planning for fall prevention in dementia. As the authors highlighted, fall risk in dementia with Lewy bodies (DLB) is multifaceted. We would like to offer a complementary perspective from the standpoint of physical rehabilitation and sensory integration.

While the study effectively demonstrated that visual dysfunction correlates with postural instability, it raises a critical question regarding the mechanism of “sensory re‐weighting.” In clinical practice, we often observe that patients with cognitive impairment struggle to switch between visual, vestibular, and somatosensory inputs [2]. A high Romberg quotient suggests a maladaptive reliance on visual input to compensate for deficits in other sensory systems. However, in DLB patients, visual processing itself is often fluctuating or impaired [3].

Therefore, simply identifying the reliance on vision is the first step. The logical next step for “care planning,” as proposed by the authors, should involve differentiating whether this visual dependence is a compensatory strategy that should be supported (e.g., by enhancing environmental lighting and visual cues) or a potential distractor that may precipitate falls when visual information is ambiguous.

We suggest that future research could benefit from combining the Romberg quotient with dynamic posturography or dual‐task gait analysis. This would help determine if rehabilitation interventions should focus on “visual cueing training” or, conversely, “sensory re‐weighting training” to reduce excessive visual dependence. The findings by Nakai et al. [1] provide an excellent foundation for tailoring such precision rehabilitation strategies.

## Funding

The authors have nothing to report.

## Ethics Statement

The authors have nothing to report.

## Consent

The authors have nothing to report.

## Conflicts of Interest

The authors declare no conflicts of interest.

## Data Availability

Data sharing not applicable to this article as no datasets were generated or analysed during the current study.
